# Metformin Can Be Safely Used in Patients Exposed to Contrast Media: A Systematic Review and Meta-Analysis

**DOI:** 10.1159/000527384

**Published:** 2022-10-06

**Authors:** Hua Qiao, Yimin Li, Bao Xu, Zhiping Lu, Jing Zhang, Danxin Meng, Shenghu He, Jin Huang

**Affiliations:** ^a^Department of Cardiology, JinTan First People's Hospital, Changzhou, China; ^b^Department of Cardiology, Sir Run Run Hospital, Nanjing Medical University, Nanjing, China; ^c^Department of Cardiology, Northern Jiangsu People's Hospital, Yangzhou, China; ^d^Department of Cardiology, Nanjing Chest Hospital, Nanjing, China

**Keywords:** Metformin, Contrast-induced acute kidney injury, Contrast medium

## Abstract

**Background:**

There have been few studies published on the use of contrast media (CM) in metformin-treated patients. In this study, we conducted a systematic review and meta-analysis to investigate the relationship between metformin and contrast-induced acute kidney injury (CI-AKI).

**Methods:**

A comprehensive search of the Medline, PubMed, Embase, and Web of Science databases for literature on associations between metformin use and CI-AKI incidence was conducted. The pooled odds ratio (*OR*), or relative risk, as well as the corresponding 95% confidence intervals (*CIs*), was calculated to assess the relationship between metformin and CI-AKI risk as well as the incidence of lactic acidosis (LA).

**Results:**

In total, seven studies met our eligibility criteria on associations between metformin use and CI-AKI incidence, comprising 2,325 individuals, with 279 new cases of CI-AKI exposed to CM. The pooled analysis revealed no statistically significant increase in the risk of CI-AKI development in patients who used metformin continuously (random-effects *OR*: 1.15, 95% *CI*: 0.70–1.90, *p* = 0.57). No cases of LA that occurred during CM exposure were reported.

**Conclusion:**

Metformin can be safely used in patients with moderate renal impairment (eGFR ≥ 30 mL/min/1.73 m^2^) during CM exposure.

## Introduction

Contrast-induced acute kidney injury (CI-AKI) has been identified to be a major healthcare problem. With the widespread use of contrast media (CM) in diagnostic and interventional procedures, CI-AKI ranks third in hospital-acquired AKI [1]. In the general population, the incidence of CI-AKI ranged from 12% to 27%, but escalated to 50% or more in patients with multiple risk factors [2]. CI-AKI deserves more attention as it has been associated with increased mortality, prolonged hospitalization, a high incidence of complications, and high treatment costs [3].

Metformin is indicated as the first-line medical treatment for type 2 diabetes mellitus (T2DM) unless there are contraindications [4]. Its anti-inflammatory and cardioprotective effects, in addition to its glucose-lowering properties, can reduce all-cause mortality [5]. Metformin has no direct nephrotoxic effect, but it is cleared via renal filtration, and very high circulating levels from an overdose or acute renal failure have been associated with lactic acidosis (LA) [6]. However, due to the extremely rare complication of LA, metformin can be safely used in patients with renal impairment. Thus, the European Medicines Agency [7] and the Food and Drug Administration (FDA) [8] amended the metformin label to highlight its safety in patients with an estimated glomerular filtration rate (eGFR) of 30 mL/min/1.73 m^2^ or greater.

There have been few studies published on the use of CM in metformin-treated patients. Early guidelines based on expert consensus have strictly recommended discontinuing metformin before using CM [9]. However, guidelines for the use of metformin and CM have become less stringent following the recent FDA recommendation update. The CMSC [10] amended their recommendations so that patients with eGFR >30 mL/min/1.73 m^2^ and no evidence of AKI can continue to take metformin normally while using CM, as the American College of Radiology (ACR) did for patients with eGFR of 30 mL/min/1.73 m^2^ or greater [11]. Nonetheless, these guidelines lacked direct evidence-based support. Thus, in this study, we conducted a systematic review and meta-analysis to investigate the relationship between metformin and CI-AKI in order to provide direct evidence of metformin use in patients with renal dysfunction who would be exposed to CM.

## Methods

### Search Strategy

This study was conducted in accordance with the protocols specified in the Preferred Reporting Items for Systematic Reviews and Meta-Analysis (PRISMA) statement (online Suppl. File S1; see www.karger.com/doi/10.1159/000527384 for all online suppl. material) [12]. A comprehensive search was conducted independently by two reviewers on the Medline, PubMed, Embase, and Web of Science databases for literature on associations between metformin and CI-AKI. To search for and retrieve all potentially relevant articles on this topic, the following MeSH terms were used: ([“metformin” OR “metformin hydrochloride” OR “metformin HCl” OR “dimethyldiguanide” OR “dimethylguanylguanidine”] AND [“contrast-induced acute kidney injury” OR “contrast-induced acute kidney injury” OR “contrast-induced nephropathy” OR “acute renal injury” OR “acute renal injuries” OR “acute kidney injury” OR “acute kidney injuries” OR “contrast media” OR “contrast agent” OR “contrast materials” OR “contrast agents” OR “contrast material” OR “radiocontrast agent” OR “radiocontrast agents” OR “radiopaque media”]). A manual search was also conducted by analyzing the reference lists of the retrieved original and review articles.

### Eligibility Criteria

In this current meta-analysis, we included observational cross-sectional, case-control, or cohort studies published between March 1, 1989, and March 1, 2020, that investigated the relationship between metformin prescription or continued use and the CI-AKI incidence in patients exposed to CM. Studies on patients with thyroid and parathyroid diseases, severe chronic kidney diseases (CKDs), or immunological diseases, as well as children, were excluded. If multiple articles from the same cohort were published, the most recent one was chosen. Renal function was classified into stages, with eGFR levels of 90 mL/min/1.73 m^2^ or higher considered as normal, 60–89 mL/min/1.73 m^2^ as mildly impaired, and <60 mL/min/1.73 m^2^ as moderately impaired.

### Data Extraction

Data from relevant studies were extracted independently by two reviewers using a standard form. Any disagreement between the two reviewers or all authors was resolved through discussion to reach a consensus. The following data were extracted: study title, first author name, publication year, baseline renal functions, participants' demographic data, number of CI-AKI cases in different groups, and number of cases with LA.

### Data Quality Assessment

The Newcastle-Ottawa Quality Assessment Scale was used to evaluate each study in terms of three domains: participant recruitment and selection; similarity and comparability between groups; and ascertainment of the outcome of interest among cohort studies [13]. Furthermore, the risk of bias in the included studies was assessed using the following criteria: (1) random sequence generation; (2) allocation concealment; (3) blinding of participants and personnel; (4) blinding of outcome assessment; (5) incomplete outcome data; (6) selective reporting; and (7) other biases.

### Statistical Analysis

To assess the relationship between metformin and CI-AKI risk, the pooled odds ratio (*OR*) or relative risk, as well as the corresponding *95%* confidence intervals (*CIs*), were calculated. The χ^2^ test, based on *Q*- and *I*^*2*^-statistics, was used to assess heterogeneity. For the Q-statistics, significant heterogeneity was defined as *p* values >0.10. The I^2^ statistic ranges from 0 to 100%, with *I*^*2*^ of <25% indicating low heterogeneity, *I*^*2*^ of 25–50% indicating moderate heterogeneity, and *I*^*2*^ >50% indicating substantial heterogeneity. The random-effects model was used for studies with substantial heterogeneity (*I*^2^ > 50%), whereas the fixed-effects model was used for studies with no substantial heterogeneity to calculate the combined *OR* values. In a subgroup analysis, the incidence of CI-AKI in metformin (+) and metformin (−) populations was compared between studies based on metformin use.

All results in this analysis were considered significant when two-tailed *p* values were found to be < 0.05. The publication bias was assessed using the *Begg's* and *Egger's* tests. All statistical analyses were performed using the *STATA* software version 16.0 (Stata Corp., College Station, TX, USA).

## Results

### Characteristics of the Included Studies

**Fig. 1:**
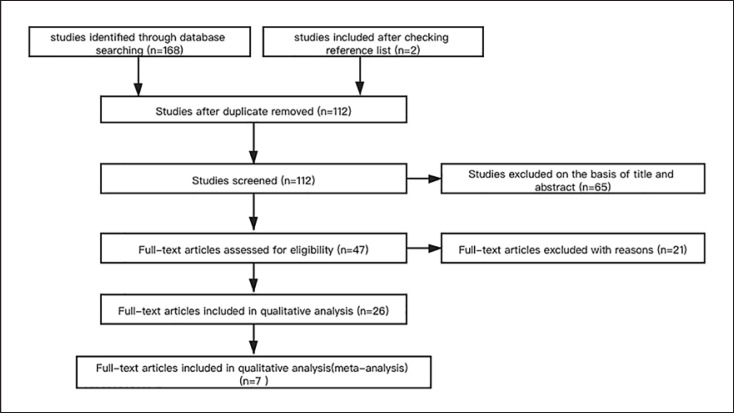
Study flowchart.

The study flowchart is shown in Figure 1. In total, 168 potentially relevant articles were identified from the initial search, and 2 additional studies were identified from the references of the retrieved articles. After the exclusion of 58 duplicated articles, the titles and abstracts of 112 articles were reviewed, and 65 additional articles were then excluded. Furthermore, the full texts of 74 articles were reviewed, and 21 articles that did not meet the eligibility criteria were excluded. Out of the 26 full-text articles reviewed, 7 studies were able to meet our eligibility criteria on associations between metformin and CI-AKI (Table 1) [14, 15, 16, 17, 18, 19, 20]. These studies comprised 2,325 individuals, with 279 new cases of CI-AKI exposed to CM. All the included studies were noted to be of high quality.

### Association between Metformin and CI-AKI

**Fig. 2:**
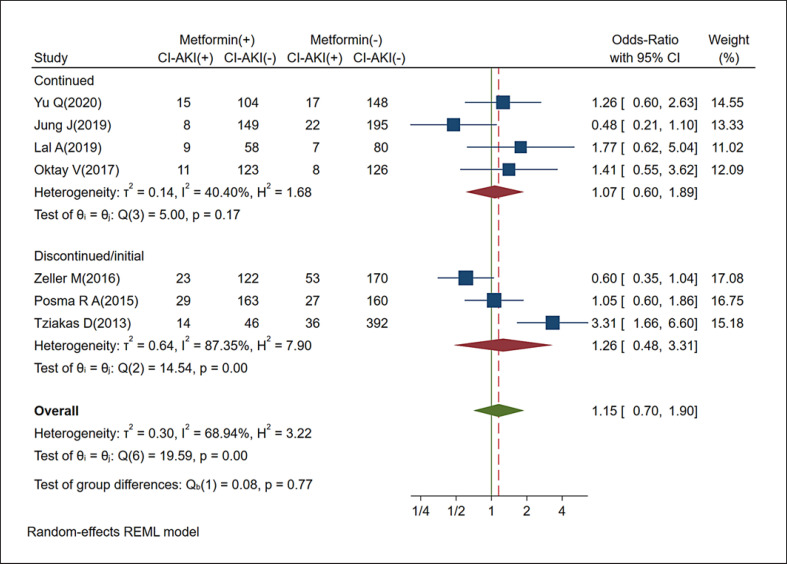
Forest plot of all studies that have examined the relationship between metformin and CI-AKI incidence (subgroup analysis based on metformin use during CM exposure).

In four of the seven studies, the increase in CI-AKI incidence was noted in patients who continued to use metformin, with statistical significance in only one study. The pooled analysis revealed no statistically significant increase in the risk of CI-AKI development in patients who used metformin continuously (random-effects *OR*: 1.15, 95% *CI*: 0.70–1.90, *p* = 0.57). In subgroup analysis based on metformin use, the CI-AKI incidence was comparable in patients exposed to CMs between those taking metformin and those who did not (continued use *OR*: 1.07, 95% *CI*: 0.60–1.89, discontinued/initial use *OR*: 1.26, 95% *CI*: 0.48–3.31; Fig. 2). These findings indicate that metformin can be used safely in patients with eGFR >30 mL/min/1.73 m^2^ who were compelled to undergo CM exposure.

### The Incidence of Metformin-Associated LA

As shown in Table 2, data on metformin-associated LA were retrieved from five of the included studies, with no cases of LA occurring in four of the articles. In another study, the serum total CO_2_ level was used as the diagnostic criterion for metabolic acidosis (MA) [15]. Nonetheless, the incidence of MA did not differ significantly between patients who took metformin and those who did not (58% vs. 65%, respectively, *p* = 0.195).

### Heterogeneity and Publication Bias

The included studies on the association between metformin and CI-AKI showed marked heterogeneity (*I*^*2*^ = 68.94%, *p* = 0.00). When subgroup analysis was conducted based on continued metformin use, heterogeneity was noted to decrease significantly (*I*^*2*^ = 40.40%, *p* = 0.17). Therefore, metformin use during CM exposure may be a critical factor in high heterogeneity.

**Fig. 3:**
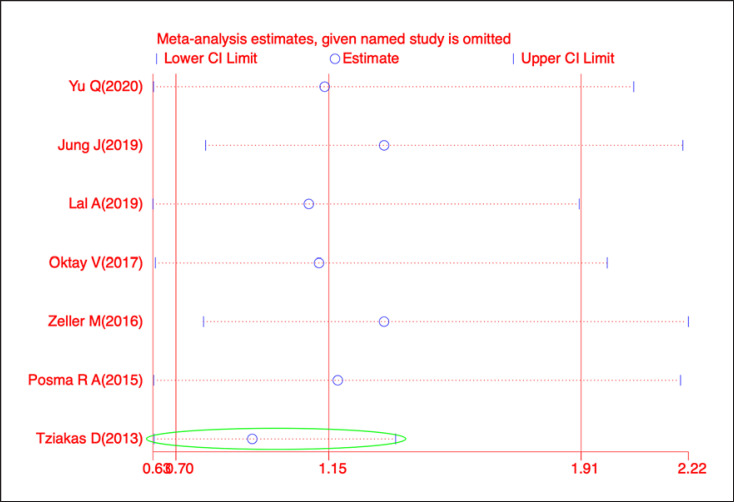
Sensitivity analysis of the relationship between metformin use and CI-AKI incidence.

**Fig. 4:**
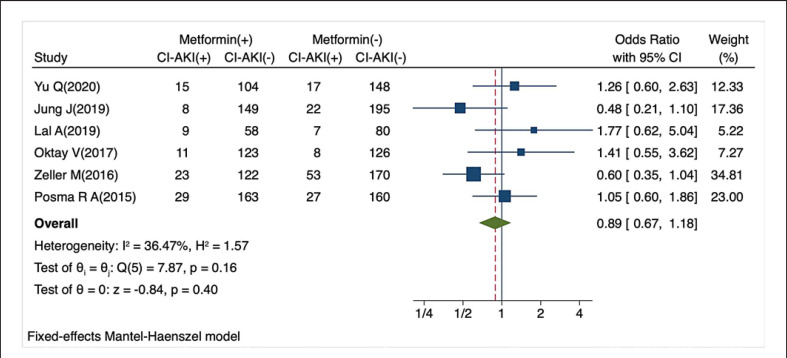
Forest plot of studies, with the exclusion of Tziakas's article, exploring the relationship between metformin use and CI-AKI incidence.

**Fig. 5:**
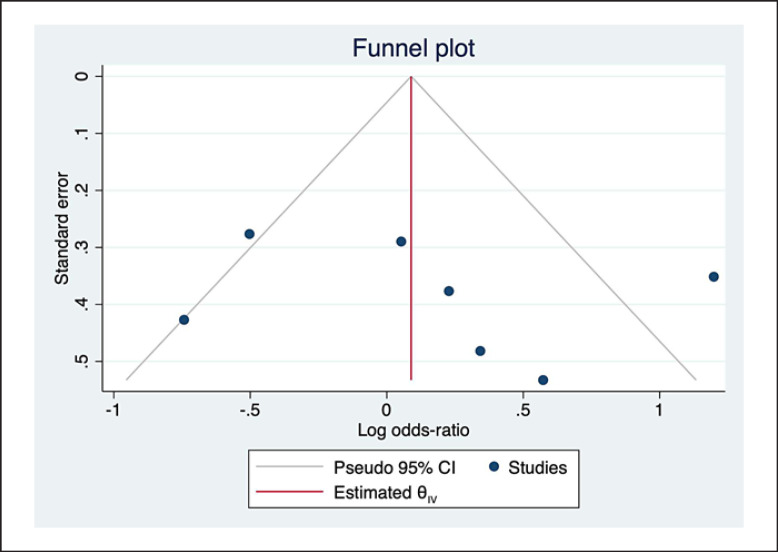
Funnel plot of the relationship between metformin use and CI-AKI incidence (*Egger*'s test: 0.5963 and *Begg*'s test: 0.2296).

As per the sensitivity analysis, the pooled ORs were unstable due to the Tziakas D' study (2013) [20] (Fig. 3). After excluding this study, the heterogeneity decreased dramatically (*I*^*2*^ = 35.04%, *p* = 0.16). Meanwhile, the pooled analysis revealed that the incidence of CI-AKI did not differ significantly between patients who took metformin and those who did not (fixed-effects OR: 0.89, 95% CI: 0.67–1.18, *p* = 0.40; Fig. 4). Visual inspection of the funnel plot revealed no obvious publication bias, and this result was supported by both the *Egger*'*s* (*p* = 0.5963) and *Begg*'*s* tests (*p* = 0.2296) (Fig. 5).

## Discussion

This systematic review and meta-analysis of 7 studies, comprising 2,325 individuals and 279 new cases of CI-AKI, have revealed no significant association between metformin use and CI-AKI incidence. Continuous metformin therapy had no deleterious effects on renal function, regardless of whether CM was administered via coronary or venous routes. To the best of our knowledge, this is the first study to provide direct evidence for the safe use of metformin during CM exposure in patients with moderate renal impairment (eGFR ≥ 30 mL/min/1.73 m^2^).

T2DM, which is considered a risk factor for coronary heart disease, has also been identified as a potential risk factor for CI-AKI [21]. According to the American Diabetes Association's latest Standards of Medical Care in Diabetes (2020) [22], metformin remains to be the first-line therapy in T2DM and would be used as monotherapy in combination with lifestyle modifications for many patients. Metformin is mainly excreted unchanged in the urine via the renal tubules, and it is cleared rapidly, with a clearance rate 3.5 times that of creatinine. Regardless of baseline renal function, plasma metformin concentration was found to be negatively correlated with eGFR [23]. Unfortunately, the therapeutic range of metformin concentration has yet to be determined [24], as it is often influenced by differences in oral metformin bioavailability, genetic variability in metformin transporters, and renal clearance [25]. Therefore, using metformin in patients with renal insufficiency is challenging.

Historically, the development of LA was a nightmare that hampered the use of metformin and the prejudice originated from phenformin, a metformin precursor. Phenformin, which was first launched in the 1950s, has been widely used before it was banned in 1977 due to the frequency of LA (40–64 cases per 100,000 people/year) and the associated mortality [26]. Unlike phenformin, the causal relationship between metformin and LA is controversial, and metformin-associated LA is reported in only 3–9 cases per 100,000 patients/year, with a mortality rate of about 50% [27]. In a nested case-control study on 50,048 patients with T2DM, 6 cases of LA were identified, yielding a crude incidence rate of 3.3 cases per 100,000 people/year with metformin therapy and 4.8 cases per 100,000 people/year with sulfonylureas [28]. The development of LA may be related to poor clinical conditions rather than specific types of oral antidiabetic medications. Meanwhile, several studies suggest that metformin has no effect on LA incidence in patients with T2DM and normal, mild, moderate, or severe renal insufficiency [29]. Metformin appeared to be safe and effective in moderate-to-severe CKD when a fixed dose was adjusted for CKD stage (1,500 mg/day in CKD stage 3A, 1,000 mg/day in CKD stage 3B, and 500 mg/day in CKD stage 4) [23].

Although nephrotoxicity is evident in all iodine CM, the association between CM use and the development of AKI remains controversial [30]. Pre-existing renal insufficiency, acute hyperglycemia, nephrotoxic drugs, and other variables were also found to be important risk factors for AKI in patients exposed to CM [31]. Due to a lack of current evidence linking metformin and CI-AKI, the European Society of Urogenital Radiology (ESUR) guideline in 1999 recommended that metformin be discontinued before CM exposure in patients with normal serum creatinine and restarted 48 h later with rechecked normal renal function [32]. The effect of metformin on renal function and LA development has been considerably overestimated as the research progressed. In 2018, the latest ESUR/CMSC guideline recommended that metformin be discontinued in patients with eGFR >30 mL/min/1.73 m^2^ and no evidence of AKI receiving either intravenous or intra-arterial iodine CM with second-pass renal exposure [9]. Regrettably, the ESUR/CMSC, ACR, and RSTN guidelines were all based on the 2016 FDA's recommendations about the safety of metformin use in patients with renal dysfunction [9], rather than findings from studies focusing on metformin performance in patients exposed to CM.

In this present study, which is the first reported meta-analysis on the exposure to both CM and metformin, 279 cases of CI-AKI were observed during CM exposure, accounting for 12% of total patients with normal to moderate renal impairment. Patients who discontinued metformin would then face the same CI-AKI risk during CM exposure as those who continued. Simultaneously, no cases of LA were detected during hospitalization except in Jung's study [15], which used MA instead of LA as the target index; however, MA could not reflect the true LA state. Our findings could have significant clinical implications. Afterward, the potential negative effects of metformin should not be considered in diabetic patients exposed to intravenous or arterial CM except for first-pass renal exposure and eGFR of 30 mL/min/1.73 m^2^ or higher, as approved by the FDA. In addition to its ability to lower blood glucose, metformin has shown substantial efficacy in lowering plasma lipids, body weight, and micro- and macrovascular events [29]. However, the only available biguanide with a pleiotropic effect on inflammatory tone and oxidative stress may affect the control of atherosclerotic plaque progression in patients with acute myocardial infarction [33]. If our concerns as regards metformin-associated LA are alleviated, patients with atherosclerotic diseases might benefit from active metformin treatment, whether they have diabetes or not. However, further large prospective clinical studies are warranted to support our findings.

This study has some limitations. First, a few related studies were included, with significant heterogeneity. Given that metformin use during CM exposure could be the key to investigating the relationship between metformin and CI-AKI, we have reluctantly performed a subgroup analysis, which was consistent with the previous results, but heterogeneity decreased sharply. Meanwhile, metformin dosage, baseline renal function, and population differences were all possible causes of the high heterogeneity. Second, sensitivity analysis revealed that Tziakas's study [20] was the main contributor to the instability of the pooled ORs. However, we chose not to exclude this study because it had no effect on the final results. Finally, due to a lack of data, further stratified analysis was deemed not possible.

## Conclusion

Based on previous research, this present study indicates that metformin can be safely used in patients with moderate renal impairment (eGFR ≥ 30 mL/min/1.73 m^2^) during CM exposure. So far, this is the only direct evidence of safe metformin use while exposed to CM.

## Statement of Ethics

All of the results and analyses were derived from previously published studies. Therefore, no ethical approval or patient consent was required.

## Conflict of Interest Statement

The authors have no conflicts of interest to declare.

## Funding Sources

This study was not funded.

## Author Contributions

Conceptualization: Qiao Hua and Li Yimin. Data curation: Qiao Hua and Xu Bao. Formal analysis: Li Yimin. Methodology and project administration: Lu Zhiping. Resources: Zhang Jing. Software: Li Yimin and Lu Zhiping. Validation: Meng Danxin. Visualization: Li Yimin and Zhang Jing. Writing − original draft: Yimin Li and Xu Bao. Writing − review and editing: Yimin Li, He Shenghu, and Jin Huang.

## Data Availability Statement

All data generated or analyzed during this study are included in this article and its online supplementary material. Further inquiries can be directed to the corresponding author.

## Supplementary Material

Supplementary data

## Figures and Tables

**Table 1:** Characteristics of the included studies on relations between metformin and CI-AKI

Reference	Publication year	Study design	Sample size	Gender (M/F)	Diagnosis	Comorbidities				CM	Exposure method	Baseline renal functions	Usage of metformina	Metformin CI-AKI (+)		Metformin (−)	
						DM	Hypertension	Dyslipidemia	Current smoking					CI-AKI (+)	CI-AKI (−)	CI-AKI (+)	CI-AKI (−)
Yu Q et al. [14]	2020	Cohort study	284	211/73	STEAMI	284	188	114	179	LOCM-IOCM	CAG	Normal to moderate impairment	Continued	15	104	17	148
Jung J et al. [15]	2019	Case-control	374	212/162	NA	374	257	NA	NA	NA	CTA	Moderate impairment	Continued	8	149	22	195
Lal A et al. [16]	2019	Case-control	154	84/70	AMI	94	NA	NA	NA	NA	CAG	Mild to moderate impairment	Continued	9	58	7	80
Oktay V et al. [17]	2017	Cohort study	268	163/105	CHD	268	225	169	77	LOCM	CAG	Normal to mild impairment	Continued	11	123	8	126
Zeller M et al. [18]	2016	Cohort study	368	NA	STEAMI	368	238	208	101	HOCM-IOCM	CAG	Normal to moderate impairment	Stopped after PCI	23	122	53	170
Posma R A et al. [19]	2015	RCT	379	284/95	STEAMI	379	112	239	209	LOCM	CAG	Normal	Taken within 3h after PCI	29	163	27	160
Tziakas D et al. [20]	2013	Cohort study	488	360/128	CHD	154	282	192	166	LOCM	CAG	Normal to moderate impairment	Withheld for 48 h prior	14	46	36	392

STEAMI, ST elevated acute myocardial infarction; AMI, acute myocardial infarction; CHD, coronary heart disease; DM, diabetes mellitus; LOCM, low osmolar contrast media; IOCM, iso-osmolar contrast media; HOCM, high osmolar contrast media; CAG, cardioangiography; CTA, CT angiography. aThe usage of metformin during the exposure to CM in observation group.

**Table 2:** The incidence of LA during CM exposure

Reference	Publication year	Diagnostic criteria of LA	Metformin (+)		Metformin (−)	
			LA (+)	LA (−)	LA (+)	LA (−)
Yu Q et al. [14]	2020	Lactate >5 mmol/L and pH <7.35	0	119	0	165
Jung J et al. [15]	2019	Total CO2 level <23 mmol/L^a^	91	66	141	76
Lal A et al. [16]	2019	NA	NA	NA	NA	NA
Oktay V et al. [17]	2017	Lactate >5 mmol/L and pH <7.35	0	134	0	134
Zeller M et al. [18]	2016	Lactate >5 mmol/L and pH <7.35	0	147	0	225
Posma R A et al. [19]	2015	Lactate >5 mmol/L and pH <7.35	0	192	0	187
Tziakas D et al. [20]	2013	NA	NA	NA	NA	NA

a MA, metabolic acidosis; LA, lactic acidosis.
